# Proposed new mechanism for food and exercise induced anaphylaxis based on case studies

**DOI:** 10.1186/1710-1492-9-11

**Published:** 2013-03-20

**Authors:** Jennifer Yan Fei Chen, Jaclyn Quirt, Kihyuk Jason Lee

**Affiliations:** 1 Queen’s University, 99 University Avenue, Kingston, ON, K7L 3N6, Canada; 2 Internal Medicine Resident, Hamilton Health Sciences, McMaster University, Hamilton, ON, Canada; 3 Division of Allergy and Clinical Immunology, St. Michael’s Hospital, University of Toronto, Toronto, ON, Canada

**Keywords:** Food and exercise-induced anaphylaxis, Food allergy, Exercise, Anaphylaxis, Mechanism

## Abstract

We present two cases of food and exercise-induced anaphylaxis (FEIA) in patients with a diagnosis of oral allergy syndrome (OAS) to the implicated foods. Patient A had FEIA attributed to fresh coriander and tomato and Patient B to fresh celery. These food allergens have been implicated in OAS and have structural antigenic similarity to that of birch and/or grass. Both patients’ allergies were confirmed by fresh skin prick tests. In both cases, strenuous exercise was antecedent to the systemic anaphylaxis reaction and subsequent ingestion without exercise produced only local symptoms of perioral pruritus. We review the current proposed mechanisms for food and exercise induced anaphylaxis to oral allergens and propose a novel and more biologically plausible mechanism. We hypothesize that the inhibitory effects of exercise on gastric acid secretion decreases the digestion of oral allergens and preserves structural integrity, thereby allowing continued systemic absorption of the allergen whether it be profilins, lipid transfer proteins, or other antigenic determinants.

## Background

Food and Exercise-induced anaphylaxis (FEIA) is a variant of exercise-induced anaphylaxis (EIA) that was first described by Maulitz et al. in 1979 [1]. FEIA differs from typical EIA in that ingestion of food allergens is required prior to exercise in order to elicit an IgE-mediated anaphylactic reaction [2]. Food allergen or exercise alone cannot elicit the anaphylactic reaction in FEIA and as such both factors are required. FEIA can occur in both children and adults. The exact prevalence of this rare phenomenon is unknown, but one study of Japanese junior high students revealed a 0.017% frequency of FEIA [2,3]. Clinical presentation can include dermatologic manifestations, such as flushing, urticaria and angioedema, as well as upper airway obstruction, abdominal pain, fatigue and syncope [4]. FEIA can result from ingestion of foods implicated in Oral Allergy Syndrome (OAS) such as tomatoes, celery, strawberry, wheat, and peaches [5].

OAS consists of a constellation of oral and pharyngeal symptoms that appear shortly after the ingestion of foods. This reaction can be due to food proteins cross-reacting with inhalant allergens [6]. Symptoms range from a spectrum of pruritus and limited lip, tongue, face or palate angioedema to angioedema of the throat and anaphylaxis. The three-dimensional structural integrity of these protein allergens are required to elicit specific IgE-mediated mast cell degranulation. Some protein allergens are digestion-labile, often losing their conformation after gastric acid digestion and proteolysis, consequently becoming unable to elicit IgE-mediated reactions [6].

Here we present two cases of FEIA in patients with diagnosis of OAS to the offending foods. Based on these cases and an extensive review of the literature, we propose a novel and more biologically plausible mechanism for this phenomenon.

## Case presentations

Patient A, a 32 year old female, ingested a salad containing carrots, tofu, ginger, avocado, tomato, cucumber, sesame seeds and coriander dressing. The patient then began running. Within 30 minutes of ingestion and 15 minutes after initiation of running she developed dysphagia and stomach cramping, followed within 60 minutes of ingestion by throat tightness, generalized pruritus, head and neck hives. The patient self-administered diphenhydramine at the time of reaction. She did not present to the emergency department. The entire event lasted approximately 2 hours with no recurrence of symptoms. She has since ingested the aforementioned foods multiple times individually without systemic symptoms. Patient A had a past medical history significant for spring and summer allergic rhinoconjunctivits with asthma.

The skin prick test to the inhalant allergens for patient A revealed positive reactions to dust mites, birch, cat, alder, cockroach, grass mix, and tree. While prick testing for commercial tomato extract was negative, both fresh coriander and tomato testing were positive.

Patient B, a 28 year old female, reported ingestion of celery, peanut butter, turkey breast, almond, egg, tomato, and green pepper. She subsequently began running 10–20 minutes after ingestion. Approximately 10–15 minutes after she began running she experienced acute urticaria, generalized facial swelling and tongue angioedema. This progressed to dysphagia, shortness of breath, chest tightness, wheeze, palpitations, abdominal cramping, and syncope. On presentation to the emergency department her systolic blood pressure was 70 mmHg. Treatment in hospital consisted of epinephrine, diphenhydramine and prednisone. Like patient A, she has since ingested the above foods without recurrence of symptoms on multiple occasions while experiencing only transient perioral pruritus. Symptoms resolved within 30 minutes after the epinephrine administration and did not recur. Patient B had a past medical history significant for seasonal allergic rhinitis with peaks in spring and summer.

Skin prick testing for patient B to the inhalant allergens revealed positive reactions to ragweed, grass, dust mites, cats, and tree mix while birch pollen and testing with commercial food extracts were negative. Her only positive reaction was to fresh celery skin prick.

Both patients denied medication changes, NSAID or antacid use prior to the reaction. Neither had known history of latex allergy or latex exposure on the day in question. There were no other cofactors involved including concomitant infections.

Based on the above history and testing, the diagnosis of food and exercise-induced anaphylaxis (FEIA) was made, Patient A secondary to coriander and tomato and Patient B to celery. Theses foods are implicated in OAS, with structural antigenic similarity to that of birch and/or grass [6]. This diagnosis is supported by the fact that both patients were able to tolerate the offending foods without exercise.

## Proposed mechanism for FEIA

Despite much interest and research, the exact pathophysiological mechanisms of FEIA have yet to be elucidated. Multiple hypotheses have been investigated. Barg *et al*. postulate that exercise induces a transient serum hyperosmolality, thereby increasing histamine release and resulting in FEIA after allergen exposure [7]. They demonstrated increase in histamine release from the basophils of a FEIA patient in a buffer of 340 mOsM, but this was not seen at lower osmolality. A serious criticism to this theory is that such high osmolality is pathological, since the normal range is between 280-290 mOsm [8,9]. Even with vigorous exercise, for example greater than 50 km of running, such an osmolality would never be reached [10]. Therefore, the transient increase in plasma osmolality due to exercise is unlikely sufficient to influence IgE-mediated mast degranulation.

Exercise induced effects on gastrointestinal permeability have also been postulated as a mechanism of FEIA, as food allergens cross the gastric epithelium to access the gut-associated mucosal system [11]. This theory has been disproven by the findings of Gisolfi *et al*. who demonstrated that mild to moderate cycling did not significantly change intestinal absorption after exercise [12]. Additionally, Nieuwenhoven *et al*. found that after 90 minute cycling trials at 60-70% V_O2max_, the lactulose/rhamnose ratio actually decreased significantly when compared to rest trials [13]. The trend of decreasing absorption is also confirmed by the decrease in 3-OMG/rhamnose ratio when compared to rest trials. This can be interpreted as a decrease in paracellular transport of large molecules across the intestinal epithelium, and extrapolated to food allergens. In addition Nieuwenhoven *et al*. found no difference in intestinal glucose absorption between rest and exercise at 70% V_O2max_[14].

A third hypothesized mechanism for FEIA postulates exercise-induced increases in tissue transglutaminase causing IgE antibodies to cross-link and thus enabling them to more efficiently elicit mast cell degranulation [15]. Contracting skeletal muscles have been shown to increase interleukin-6, an inflammatory mediator, that upregulates the expression of tissue transglutaminase [16,17]. However, Robson-Ansley *et al*. found that 90 minutes of vigorous exercise increased the interleukin-6 level by only 3-fold when compared to rest. Additionally, carbohydrate ingestion during exercise had a dampening effect on this increase [18]. It is therefore unlikely at typical FEIA intensity for this mechanism alone to be sufficient.

Here we propose a mechanism for FEIA to OAS foods related to suppression of gastric acid secretion due to postprandial exercise thereby decreasing digestion. A decrease in gastric acid secretion will consequently reduce digestion of some oral allergens. This may be particularly relevant in clinically relevant allergens such as lipid transfer proteins that are already somewhat resistant to digestion [19,20]. This may allow increased absorption of structurally intact allergens and may cross the threshold for IgE-mediated mast cell degranulation. For example, a recent paper identifies Api g 2, a novel allergenic member of the lipid transfer protein 1 family from celery that is particularly resistant to digestion [20]. Moreover it is important to note that the quality and quantity of gastric acid and gastric digestive enzymes are paramount in determining which allergens persist [19].

Early studies by Crandall in dogs looking at postprandial exercise have found that gastric acid secretion is suppressed during the exercising period and only starts to rise gradually after cessation [21]. Furthermore, the ascent was slower when exercise was sustained for a longer duration. The maximum acid secretion in exercise never reached that of the resting control. This suppression of gastric acid production with postprandial exercise has been replicated in many more recent studies. Konturek *et al*. found that exercise sharply reduced the secretion of gastric acid stimulated by feeding and rose in compensation after the cessation of exercise, but to no more than 65% of the peak in control trials [22].

The inhibition of gastric acid during exercise can be partly attributed to an increase in sympathetic innervation leading to increased catecholamine release. Catecholamines are partially responsible for inhibiting gastric acid production [23]. This is supported by the concurrent onset of increased heart rate and decreased acid production with escalating difficulty of exercise [24]. Aminopyrine clearance, which is used to measure mucosal blood flow in the gut, is also found to be decreased by 50% during exercise compared to resting controls. The levels increase gradually after the cessation of exercise, but never back to the rate before exercise [22]. Furthermore, Qamar and Read also demonstrated a diminished mesenteric blood flow increase with food and exercise compared to food alone [25]. The preferential perfusion of skeletal muscle that occurs during exercise secondary to sympathetic drive may not only affect gastric acid secretion as described above, but may act to increase exposure of structurally intact allergen to phenotypically different mast cells in skeletal muscle which may have a lower threshold for degranulation [26].

We propose a mechanism for FEIA involving decreased gastric acid production and thereby decreased digestion of allergens due to exercise, leading to more structurally intact allergens absorbed across the epithelial barrier. This proposed mechanism is strengthened by a study by Morita *et al*. demonstrating the presence of undigested immunoreactive wheat Ω-5 gliadin in patient sera during a wheat-and-exercise challenge but absence of such gliadin during wheat or exercise-only challenges [4]. Taken together this suggests exercise induced absorption of the undigested forms of wheat gliadin occurred, as is confirmed by the onset of allergic symptoms when the immunoreactive gliadins appeared in wheat-sensitive patients during these wheat-and-exercise challenges [4]. In addition the more recent in vitro work on digestion of allergens would also complement our proposed mechanism [19].

Finally, and convincingly, our proposed mechanism is further supported by work by Untersmayr *et al*. in mice. This group demonstrated that when digestion-labile allergens are added together with acid-suppression drugs, such as H2-receptor blockers, or proton pump inhibitors (PPIs), the allergens induced specific IgE antibodies and positive mucosal and skin reactions [27]. Even further supporting our hypothesis is that 3 months of H2-receptor blockers or PPI therapy in dyspeptic patients induced de novo IgE formation in 25% of the patients. Sensitization was seen on skin testing 5 months after cessation of antacid medications [28]. Sensitization was accompanied by IgE mediated symptoms in these patients.

## Conclusions

We propose a more biologically plausible mechanism for FEIA with OAS foods involving postprandial exercise induced gastric acid inhibition via increased sympathetic innervation and decreased mesenteric blood flow, ultimately resulting in reduced digestion of labile allergens. This may allow absorption of structurally intact proteins potentially crossing the threshold of IgE-mediated mast cell degranulation. We welcome further studies to confirm our hypothesis.

## Consent

Written informed consent was obtained from the patient for publication of this Case report and any accompanying images. A copy of the written consent is available for review by the Editor-in-Chief of this journal.

## Abbreviations

FEIA: Food and exercise-induced anaphylaxis; OAS: Oral allergy syndrome; NSAIDs: Non-steroidal anti-inflammatory drug; IgE: Immunoglobulin E; 3-OMG: 3-O-methyl-glucose.

## Competing interests

The authors declare that they have no competing interests.

## Authors’ contributions

JC conducted the literature review, drafted the manuscript, and has given final approval of the version to be published. JQ was involved with revisions and editing of the manuscript as well as verifying the literature review. JL conceived of this case report, revised it critically for intellectual content and has given final approval of the version to be published. All authors read and approved the final manuscript.
